# On the road to genomically defining bacterial intra-species units

**DOI:** 10.1128/msystems.00584-24

**Published:** 2024-06-28

**Authors:** Santiago Castillo-Ramírez

**Affiliations:** 1 Programa de Genómica Evolutiva, Centro de Ciencias Genómicas, Universidad Nacional Autónoma de México, Cuernavaca, México; Institut de Recherche pour le Developpement Delegation Regionale Occitanie Centre de Documentation, Montpellier, France

**Keywords:** intra-species units, average nucleotide identity (ANI), genomic epidemiology, strain, sequence type, evolutionary genomics

## Abstract

Over almost three decades, average nucleotide identity (ANI) analysis has been instrumental in operationally defining species in bacteria. However, barely any attention has been paid to soundly defining intra-species units employing ANI analyses until recently. Notably, some very recent publications are good steps forward in that direction. The level of granularity provided by these intra-species units will be relevant to understanding the eco-evolutionary dynamics and transmission of bacterial lineages and mobile genetic elements, antibiotic resistance, and virulence genes. These intra-species units will undoubtedly advance the genomic epidemiology of many bacterial pathogens. In the coming years, we anticipate that many studies will implement ANI-based definitions of different intra-species units, such as strains or sequence types, for many different bacterial species.

## COMMENTARY

For centuries, the taxonomic rank of species has been regarded as a fundamental unit of diversity in biology. Due to the significant differences in genetics and other biological aspects, species definitions for bacteria have differed from those used for Eukaryotes. Considering the operational definition of “species” (the actual set of rules and parameters to describe and identify species), whole genome sequencing has been a game changer when it comes to bacteria. In this regard, average nucleotide identity (ANI) analyses have been instrumental for conducting genomic coherence when circumscribing members of a bacterial species (1, 2). In particular, bacterial genomes of the same species usually exhibit ANI values of ≥96% among them; this approach has been used for almost three decades now. However, until very recently, ANI has rarely been used to define intra-species units. Of note, beyond basic microbiology research, defining intra-species units could have important clinical implications, especially when considering human and animal bacterial pathogens. For instance, particular intra-species units could have important phenotypic traits such as multidrug resistance or hypervirulence.

Considering the operational definitions of intra-species subunits when dealing with bacterial genomes, there have been two major strategies. One strategy has been the use of pregenomic typing approaches such as multilocus sequence typing (MLST), where genomes are usually assigned to sequence types (ST) already described in MLST databases (3, 4). Although this strategy allows us to put the genomes in the context of the known diversity of the species, the resolution provided by MLST (usually based just on seven loci) is far lower compared with what can be achieved with whole genome sequences. Furthermore, MLST might produce inaccurate genotyping for bacterial species with highly dynamic genomes (5). Lately, MLST schemes have been expanded to whole genome (wgMLST) and core genome (cgMLST) schemes (6).

Although, ideally, these are good tools to define intra-species units genomically—for instance, genomovars (different genomics groups in a species with similar phenotypes)—some limitations affect cgMLST and wgMLST. First, there is not a set (or at least agreed) threshold for the number of loci differences to delineate the clusters (units), not even within studies analyzing the same species. Thus, clusters found in the different studies are not equivalent nor comparable. Second, regarding the cgMLST, there is the issue of the ever-changing core genome. The core genome depends on the number (and divergence) of the genomes included. As one adds or subtracts genomes, the core is bound to change. Thus, studies would have very different cgMLSTs if they were to deal with local populations versus the whole species. The other strategy is to conduct a population structure analysis on (usually) the core genome of the isolates under consideration. These have been conducted for important pathogens such as *Acinetobacter baumannii* (7) and *Staphylococcus aureus* (8) but also with vector-borne bacteria such as *Borrelia burgdorferi* (9). One of the issues of this strategy is that the number of clusters will depend on the genetic diversity within the sampling data set; another issue is that the clusters found for the different data sets are not comparable. In this regard, an ANI-based definition of intra-species units, whichever the unit is (strain, sequence type, genomovar, etc.), can circumvent the issues of the two main strategies outlined above.

In less than 6 months, three articles have advanced and provided novel insights into the use of ANI to define intra-species units (10–12). In a recent study analyzing 330 bacterial species, the authors have shown that a well-marked aggregation of ANI values exist in isolates from individual species (10). The ANI values concentrated around a value of 99.5% (range from 99.2 to 99.8%), and this aggregation corresponds with what has been identified as STs in previous studies. In another study to expressly advance the definition of strain, the authors employ a collection of genomes from the species *Salinibacter ruber* sampled from salterns in two Spanish islands (11). Again, analyzing the distribution of ANI values for members of the species, they established an ANI value of ≥99.99% (with a shared gene content of >99.0%) to circumscribe strains. In an even more recent paper published in *mSystems*, Raghuram and colleagues (12) went one step further. Not only did they define intra-species units but used these to examine the distribution of gene families for the important nosocomial pathogen *S. aureus*. In what might be the largest study that has conducted intra-species clustering for a bacterial species, the authors process over 84,000 genomes and conduct clustering at the strain level. Then they use this clustering to better appreciate the frequency distribution of individual gene families to an unprecedented level. This study is a clear example of the ultra-resolution level that can be implemented when considering intra-species diversity. From a methodological standpoint, this study defines an approach to deal with huge bacterial genomic data sets and is bound to be a reference point for future studies implementing intra-species clustering for bacterial species.

Clearly, the appropriate ANI values to define the different intra-species units (strain, ST, etc.) for the different bacterial species will depend on the patterns of genomic variation of each species, in particular the level of homologous and non-homologous recombination. Considering this, ideally, one first needs to analyze the distribution of ANI values among the isolates of the species and from there see which ANI value thresholds are more suitable for the intra-species unit considered in the context of bacterial species under study (1, 10). The definitions of intra-species units are bound to be instrumental in appraising genomic diversity at very short microevolutionary scales, as they will add a level of granularity hardly seen before. Importantly, this will be relevant not only to understanding the dissemination (an eco-evolution) of bacterial lineages but also to comprehend the dispersion (an again eco-evolution) of mobile genetic elements, virulence, and antibiotic resistance genes.

These days, there are enormous genome data sets for many bacterial species for which it would be extremely useful to conduct intra-species analysis soundly. I anticipate that in the coming years, we will see the rise of ANI-based definitions of intra-species units and this will undoubtedly move forward the genomic epidemiology of bacteria, pathogens, and non-pathogens alike.
